# Cross dual-microcomb dispersion interferometry ranging

**DOI:** 10.1126/sciadv.adt4252

**Published:** 2025-08-15

**Authors:** Yang Wang, Jindong Wang, Jingsheng Huang, Weiqiang Wang, Long Huang, Wen Shao, Minhui Cheng, Brent E. Little, Sai Tak Chu, Wei Zhao, Wenfu Zhang

**Affiliations:** ^1^ State Key Laboratory of Ultrafast Optical Science and Technology, Xi’an Institute of Optics and Precision Mechanics, Chinese Academy of Sciences, Xi’an 710119, China.; ^2^ University of Chinese Academy of Sciences, Beijing 100049, China.; ^3^ Key Laboratory of Optoelectronic Technology & Systems (Ministry of Education), Chongqing University, Chongqing 400044, China.; ^4^ CNPC Research Institute of Safety and Environment Technology, Beijing 102206, China.; ^5^ School of Electronic Information and Artificial Intelligence, Shaanxi University of Science and Technology, Xi’an 710021, China.; ^6^ Department of Physics, City University of Hong Kong, Hong Kong, China.

## Abstract

Soliton microcombs offer unprecedented laser sources for high-precision ranging due to their merits of high repetition rate, excellent coherence, and compact size. However, high repetition rate limits the nonambiguity range (NAR) of ranging. Previous dual-comb–based methods can extend the NAR, but asynchronous measurement error (AME) is commonly introduced, which greatly limits the ranging accuracy. Here, we propose a cross dual-microcomb absolute ranging scheme based on dispersion-interferometry method. The AME introduced during dynamic measurement is completely eliminated by one-shot spectral sampling, while the NAR is extended from 3 millimeters to 339 meters by Vernier effect when the repetition-rate jitter is 2 hertz. In addition, the excellent system performance has been verified at different distances and the Allan deviation down to 5.63 nanometers after averaging 56 seconds. Our scheme boasts the potential of straightforward chip architecture and minimal detector requirements and provides an advanced method for future high-precision long-distance ranging and miniaturized lidar systems.

## Teaser

A cross dual-microcomb ranging scheme eliminates asynchronous measurement errors prevalent in previous dual-comb methods.

## INTRODUCTION

High-accuracy and long-range absolute distance measurement plays a crucial role in various fields, including aerospace, industrial production, daily life, and scientific research (*1*). The length unit “meter” is defined as the transmission distance of light in a vacuum within 1/299792458 s, which achieves the unity of time and length (*2*, *3*). Optical frequency comb (OFC) is regarded as a stable and precise “ruler” or “timer” in both the time and frequency domains (*4*). It is a key component in realizing optical clocks (*5*–*7*) and holds broad application prospects in the field of precision ranging.

In 2000, Minoshima *et al.* (*8*) realized absolute distance measurement through the beat frequency phase shift of mode-locked lasers. The distance measurement error is within ±0.002 m at a distance of 239.943 m, which is the precursor of OFC–based precision ranging. Since then, various technologies were developed, which led to substantial advancements in precision ranging based on femtosecond OFCs (*9*–*21*). Among these ranging technologies, the dispersion interferometry (DPI) ranging method made full use of the high coherence advantage of femtosecond OFC and proved the feasibility for high-precision absolute distance measurement (*10*). Nevertheless, traditional femtosecond OFCs are usually bulk, and it is difficult to achieve comb-tooth resolution using spectrometers or grating demodulators when using them for DPI ranging. Reciprocally, the emerging soliton microcombs (SMCs) (*22*–*33*)–based microcavities have the characteristics of high repetition rates due to their small size, which can easily realize the comb-tooth resolution without the need for optical narrowband filters, completely eliminating the measurement dead zone (*34*, *35*). In addition, SMCs feature natural high coherence, enabling higher sensitivity in ranging systems by using multiple comb-teeth channels for coherent parallel measurements (*36*). These advantages make it widely used to achieve long-range, high-precision, absolute distance measurements (*34*–*42*). However, the high repetition rate leads to a small nonambiguity range (NAR). For example, the NAR corresponding to a repetition rate of several tens of gigahertz is only in the order of millimeters. Therefore, it can generally only be used to measure small vibrations or for incremental measurements. To achieve long-range absolute distance measurements, it is necessary to combine other auxiliary measurement means (*34*).

To extend the NAR of OFCs-based ranging system, synthetic wavelength interference method (*11*, *20*) is often used. However, there is a trade-off between the NAR, accuracy, speed, and system complexity. Using the Vernier effect of dual comb is also a feasible solution, but it often requires adjusting the repetition rate of the OFC (*9*, *17*, *43*), exchanging the signal comb and local oscillator comb in the dual-comb ranging system (*12*, *37*) or using a dual-comb–swept laser (*44*).

Among these approaches, there exists an important problem that is commonly overlooked: To calculate the absolute distance, secondary sampling must be performed asynchronously after changing the repetition rate or switching the roles of dual combs, which will introduce nonnegligible asynchronous measurement error (AME) when considering the real time change of the distance in actual measurement scenario. In previous dual-comb ranging studies, some only conducted static measurements or did not compare the results with those from standard rangefinders (*9*, *12*, *17*, *37*, *43*, *44*). Others performed short-range or relative measurements without leveraging the Vernier effect for extending the NAR (*18*, *38*). Therefore, finding and obtaining AME of absolute distance measurement was challenging. For static, short-range, and relative ranging applications, the impact of AME can often be disregarded. However, in most long-distance ranging scenarios, the target is usually moving, and the length of the measurement path is usually affected by environmental fluctuations, leading to substantial AME. This error is expected to be avoided by one-shot spectral sampling, but high-resolution spectroscopy will be required to achieve precise detection of the dual-comb teeth. For SMC-based ranging system, this limitation hampers the achievement of high-accuracy dynamic measurements, raises system costs, and diminishes integration potential. Therefore, the pressing issue is how to leverage the advantages of miniaturization, high repetition rates, and outstanding coherence of SMCs to achieve high precision, no dead zones, and rapid measurements, while efficiently expanding the NAR of ranging.

Here, we propose a structurally simple on-chip cross dual-microcomb (CDMC) ranging scheme, which achieves high-precision measurements while able to extend the NAR of DPI ranging more than five orders of magnitude by using Vernier effect. Crucially, the comb teeth of CDMC exhibit a cross-distribution and uniform pattern, enabling precise one-to-one synchronous measurements with photodetector (PD) arrays at low-resolution demand, which maximizes the use of comb teeth space and eliminates the AME. In the proof-of-principle experiments, two SMCs with slightly different repetition rates and staggered comb-tooth frequencies are generated. High-precision ranging is demonstrated at different distances. The SD of stepwise measurements near a round-trip distance of 7.14 m is 3.72 μm. Long-term stable precision metrology of 1 hour is achieved at the distance of 0.3 m, with a minimum Allan deviation of 5.63 nm when the averaging time is 56 s. In addition, the measurement errors and blind spots, as well as the limitation of repetition-rate jitter on NAR are analyzed. The CDMC ranging system can be miniaturized, which paves the way for broad industrial applications of precision ranging systems.

## RESULTS

### System architecture and principle

**Fig. 1.**
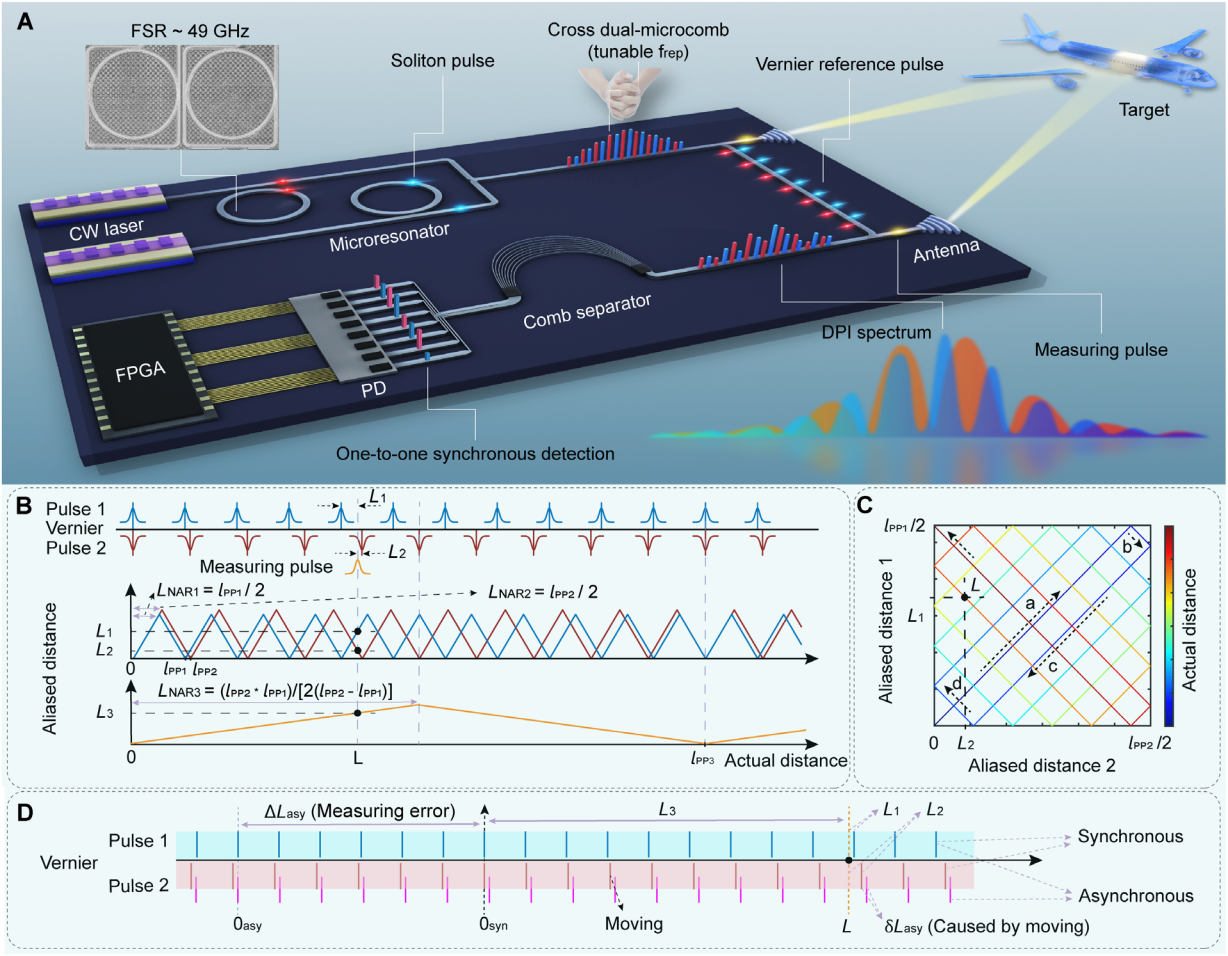
Architecture and principle of the on-chip CDMC ranging system. (**A**) Two on-chip continuous-wave (CW) lasers with a frequency difference of half the repetition-rate pump two microresonators, respectively, generating two SMCs with slightly different repetition rates and staggered tooth frequencies through Kerr nonlinear effect. The CDMC is divided into measuring (signal) and reference pulse beam. The measuring pulse illuminates the target to be measured (such as the aviation modules in assembly) through the transmitting antenna, and the reflected pulse, recovered by the receiving antenna, interferes with the reference pulse. Then, comb teeth are separated by the comb separator (such as an array waveguide grating) and synchronously detected one-to-one by the PD array. Last, the FPGA is used to calculate the absolute distance based on the Vernier effect. The upper left illustration is an image of the microring resonators, and the lower right corner illustration is the DPI spectrum diagram. (**B**) NAR expansion principle based on the Vernier effect. (**C**) The network mapping diagram between aliased distance and recovered absolute distance, where four different arrow directions correspond to four different combinations of the aliasing distance, and the total length of the trajectory is exactly the extended NAR. (**D**) Asynchronous measurement error schematic diagram. The effect of asynchronous measurement is equivalent to moving the Vernier pulse 2 by a length $\delta L_{\mathrm{asy}}$ during the second measurement. This small error causes the starting point of the Vernier pulse to move from point $0_{\mathrm{syn}}$ to point $0_{\mathrm{asy}}$ , resulting in a large error $\Delta L_{\mathrm{asy}}$ on the recovered actual distance.

The architecture of the on-chip CDMC ranging system is schematically shown in Fig. 1A. The system is composed of dual-microcomb sources, transmitting and receiving antennas, a comb separator, a PD array, and a signal process unit [such as a field-programmable gate array (FPGA)]. The two microcombs are pumped using two independent lasers, the frequency difference of which is approximately half the microcomb repetition rate. The comb teeth of the SMCs are staggered like two folded hands, and the repetition rates of the SMCs can be tuned by the thermal effect of the microcavities (as illustrated in top left corner). The CDMC is split into a measuring (signal) beam and a reference beam. The measuring beam illuminates the target (i.e., the aviation modules in assembly shown in Fig. 1A) through the transmitting antenna, and the reflected optical field is converged into the receiving waveguide by the receiving antenna. A comb separator (such as an array waveguide grating) is used to demultiplex the comb teeth, which are then synchronously detected by a PD array. Last, the absolute distance is accurately recovered in real time by the signal process unit based on DPI ranging algorithm, and the NAR is extended through Vernier effect.

For DPI ranging system, the interference spectrum intensity of each SMC can be expressed as $I\left(\nu \right)={\langle E_{r}\left(\nu \right)+E_{m}\left(\nu \right)\rangle }^{2}=E^{2}\left(\nu \right)\left[{\alpha_{r}}^{2}+{\alpha_{m}}^{2}+2\alpha_{r}\alpha_{m}\text{cos}\left(2\pi \nu \tau \right)\right]$ , where E(ν) represents the optical field of SMC, $E_{r}\left(\nu \right)=\alpha_{r}E\left(\nu \right)$ and $E_{m}\left(\nu \right)=\alpha_{m}E\left(\nu \right)\text{exp}\left(-i2\pi \nu \tau \right)$ are optical fields in the reference and measuring arms, respectively, $\alpha_{r}$ and $\alpha_{m}$ represent the corresponding amplitude attenuation coefficients, ν represent the frequency of SMC, and τ means the time delay between the reference and measuring path. The lower right inset of Fig. 1A shows the typical DPI spectrum diagram of the CDMC, which is modulated by two cosine terms. Through data processing (see “Materials and Methods” for more details), time delay τ can be calculated, and the round-trip absolute distance L can be further obtained according to formula L=cτ/n , where c is the speed of light in a vacuum and n is the refractive index of the medium. The mismatch between the repetition rate of the OFC and the resolution of optical spectrum acquisition system can induce measurement dead zone, which can be resolved using the high–repetition rate SMC. However, the high repetition rate results in limited NAR, i.e., $L_{\mathrm{NAR}}=l_{\mathrm{pp}}/\left(2n\right)=c/\left(2n\cdot f_{\mathrm{rep}}\right)$ , where $f_{\mathrm{rep}}$ is the repetition rate and $l_{\mathrm{pp}}$ is the pulse interval of the SMC in vacuum, $l_{\mathrm{pp}}=c/f_{\mathrm{rep}}$ . In the air medium ( n≈1 ), the $L_{\mathrm{NAR}}$ corresponding to the repetition rate of 50 GHz is only 3 mm, while distance aliasing will occur when the measured distance is greater than 3 mm. For a long-distance measurement, the round-trip absolute distance L under test will contain both an integer and a fraction part and can be expressed as $L=\left(N\cdot l_{\mathrm{pp}}\pm c\tau /n\right)$ , where L can be calculated by measuring the integer N and the flight delay time τ . Therefore, an additional ranging means, such as a dual-frequency phase-modulated laser range finder (*34*), is needed to determine the integer N.

Two SMCs with a slight difference in repetition-rate are used in the CDMC ranging system, constituting a set of Vernier pulses in temporal domain (see Fig. 1B). As the measuring distance increases, the two different fractional parts (i.e., aliased distances) $L_{1}$ and $L_{2}$ change in a triangular waveform with periods corresponding to the adjacent pulse intervals of the two SMCs, respectively, which can be obtained from the interference spectra. Using the Vernier effect, the actual distance (i.e., round-trip absolute distance) L can be further calculated on the basis of the values of $L_{1}$ and $L_{2}$ . $l_{\mathrm{pp}1}$ and $l_{\mathrm{pp}2}$ represent the adjacent pulse intervals of the two SMCs, and the corresponding NARs in vacuum are $L_{\mathrm{NAR}1}=l_{\mathrm{pp}1}/2$ and $L_{\mathrm{NAR}2}=l_{\mathrm{pp}2}/2$ , respectively. As shown in Fig. 1B, except for the zero point, the upper (pulse 1) and lower (pulse 2) sets of the Vernier pulses will align again at a longer distance that corresponds to the Vernier pulse repetition period $l_{\mathrm{pp}3}$ . The extended NAR is half of this period. On the basis of the Vernier effect, we can easily get relationship $l_{\mathrm{pp}3}=ml_{\mathrm{pp}2}=\left(m+1\right)l_{\mathrm{pp}1}$ , where m is a positive integer. Therefore, the extended NAR is derived as

$$L_{NAR3}=l_{pp3}/2=\left(l_{pp2}\cdot l_{pp1}\right)/\left(2\left(l_{pp2}-l_{pp1}\right)\right)=c/\left(2∆f_{rep}\right)$$ (1)

where $∆f_{rep}=f_{rep1}-f_{rep2}=c/l_{pp1}-c/l_{pp2}$ . It can be seen that the extended NAR is no longer inversely proportional to the repetition rate $f_{\mathrm{rep}}$ , but to the repetition rate difference $∆f_{rep}$ , which means that the original aliasing distance can be recovered.

By plotting the above two groups of triangular waves on the horizontal and vertical coordinates, respectively, the network mapping diagram (Fig. 1C) between aliased distance and actual distance can be obtained, where each two-dimensional coordinate point corresponds to a set of ( $L_{2}$ , $L_{1}$ ) values. The actual distance starts at the coordinate (0, 0) and rotates clockwise along the arrow directions a , b , c , and d until it reaches the end point (0, $l_{\mathrm{pp}1}/2$ ). The total length of the trajectory is exactly the extended NAR. As the actual distance increases, both $L_{1}$ and $L_{2}$ increase or decrease periodically, resulting in four different combinations of aliased distance: ( $L_{2}↑$ , $L_{1}↑$ ), ( $L_{2}↑$ , $L_{1}↓$ ), ( $L_{2}↓$ , $L_{1}↓$ ), and ( $L_{2}↓$ , $L_{1}↑$ ). These combinations correspond to the above four different arrow directions, where the symbols ↑ and ↓ represent increasing and decreasing, respectively. According to the simple geometric relationship, the distance recovery formula corresponding to the above four combinations can be obtained as

$$\begin{array}{c} \left\{\begin{array}{c} \begin{array}{c} \;L_{a}=N_{a}l_{\mathrm{pp}1}+L_{1}=N_{a}l_{\mathrm{pp}2}+L_{2}\\ \;L_{b}=\left(N_{b}+1\right)l_{\mathrm{pp}1}-L_{1}=N_{b}l_{\mathrm{pp}2}+L_{2\;} \end{array}\\ \begin{array}{c} \;L_{c}=\left(N_{c}+1\right)l_{\mathrm{pp}1}-L_{1}=\left(N_{c}+1\right)l_{\mathrm{pp}2}-L_{2}\\ \;L_{d}=\left(N_{d}+1\right)l_{\mathrm{pp}1}+L_{1}=\left(N_{d}+1\right)l_{\mathrm{pp}2}-L_{2} \end{array} \end{array},\begin{array}{c} \;\begin{array}{c} \left(a\right)\\ \left(b\right) \end{array}\\ \begin{array}{c} \;\left(c\right)\\ \;\left(d\right) \end{array} \end{array}\right\} \end{array}$$ (2)

where, $L_{i}$ and $N_{i}$ ( i=a,b,c,d ) are the actual distance and the number of adjacent pulse intervals $l_{\mathrm{pp}1}$ contained in the four cases, respectively. For an unknown actual distance less than $L_{\mathrm{NAR}3}$ , considering the uniqueness of the solution, only one of the four possible values $L_{a}$ , $L_{b}$ , $L_{c}$ , and $L_{d}$ calculated according to Eq. 2 satisfies the condition $0<L_{i}<L_{\mathrm{NAR}3}$ , and this solution is the recovered actual distance L . In an actual measurement scenario, atmospheric turbulence and the movement of the measured target often lead to the real-time changes in the measured distance. Therefore, there is a serious and widespread problem: If the two fractional parts $L_{1}$ and $L_{2}$ in Eq. 2 are measured asynchronously, large AME will be introduced. As shown in Fig. 1D, the effect of asynchronous measurement is equivalent to moving the Vernier pulse 2 by a length $\delta L_{\mathrm{asy}}$ during the second measurement. Because of the magnification of the Vernier effect, this small error $\delta L_{\mathrm{asy}}$ caused by the ruler movement further causes the starting point of the Vernier effect (where the upper and lower Vernier pulse scales are aligned) to move from point $0_{\mathrm{syn}}$ to point $0_{\mathrm{asy}}$ , resulting in a large error $\Delta L_{\mathrm{asy}}$ on the recovered actual distance. Taking Eq. 2 (a) as an example for quantitative analysis, its expression becomes $L_{a}+\Delta L_{\mathrm{asy}}=N_{a}l_{\mathrm{pp}1}+L_{1}=N_{a}l_{\mathrm{pp}2}+L_{2}+\delta L_{\mathrm{asy}}$ , and after deriving, we can get the relation $\Delta L_{\mathrm{asy}}=-\delta L_{\mathrm{asy}}\cdot l_{\mathrm{pp}1}/\left(l_{\mathrm{pp}2}-l_{\mathrm{pp}1}\right)$ . Through further analysis, the general error formula of the recovered actual distance is

$$\Delta L_{asy}=\pm |l_{pp1}/\left(l_{pp2}-l_{pp1}\right)|\cdot \delta L_{asy}=\pm |f_{rep2}/∆f_{rep}|\cdot \delta L_{asy}$$ (3)

where the positive sign “+” is taken for Eq. 2 (a and b), and the negative sign “−” is taken for Eq. 2 (c and d). It can be seen that the error $\Delta L_{\mathrm{asy}}$ is proportional to $\delta L_{\mathrm{asy}}$ , and it is amplified $|f_{\mathrm{rep}2}/∆f_{\mathrm{rep}}\;|$ times by the Vernier effect. Taking the parameter settings ( $f_{\mathrm{rep}2}$ ~9.36 GHz, $∆f_{\mathrm{rep}}$ ~5.685 kHz) in (*37*) as an example, if the distance to be measured changes by 1 μm before the secondary sampling, the actual distance calculated according to the Vernier effect will produce a huge error of about 1.65 m when the error caused by all other factors are ignored. Fortunately, this error can be completely eliminated by the synchronous measurement method used in the CDMC ranging scheme.

### Experimental device

**Fig. 2.**
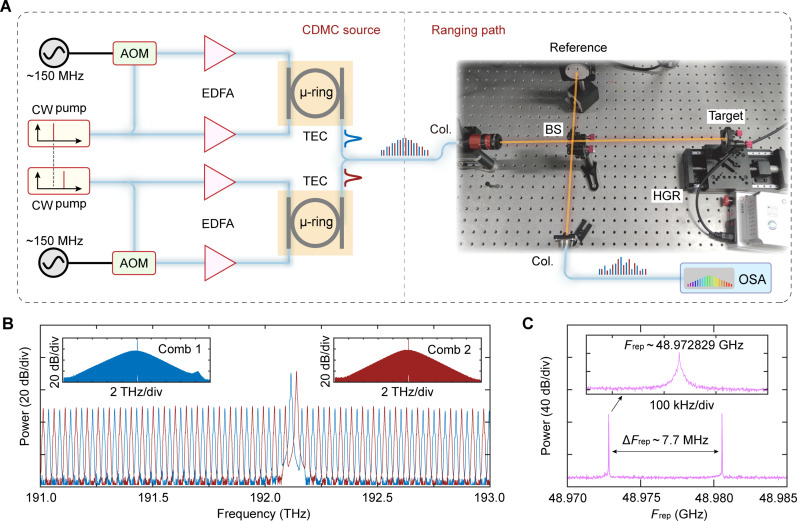
Experimental setup of the CDMC ranging. (**A**) The experimental setup diagram of on-chip CDMC ranging based on DPI. (**B**) The spectra of the two cross single SMCs. (**C**) The repetition rates are all about 49 GHz, with a difference of ~7.7 MHz, and can be adjusted within the range of 0 to 30 MHz. CW, continuous wave; AOM, acousto-optic modulator; EDFA, erbium-doped fiber amplifier; TEC, thermoelectric cooler; μ-ring, micro-ring resonator; Col., collimator; BS, beam splitter; HGR, horizontal guide rail; OSA, optical spectrum analyzer.

We designed a series of experiments to verify the performance of the CDMC ranging scheme, and the setup is shown in Fig. 2. To obtain two SMCs with slightly different repetition rates, two independent microring resonators are used, which are fabricated on high-index doped silica glass process platform. The well-developed laser-assisted intracavity thermal balance scheme is used to deterministically generate single SMCs (*31*). A tunable narrow linewidth (100 Hz) laser serves as the pump. The auxiliary laser is generated by shifting the pump frequency by ~150 MHz using an acousto-optic modulator, ensuring that the beat frequency of the pump and auxiliary waves is naturally locked, thereby improving the stability of the SMC state and reducing the jitter of the repetition rate (*32*). By thermally tuning the microcavity resonance, when the pump light is located at the red-detuning of the microcavity resonance, the mode-locked soliton state OFC is generated. Single SMCs can be formed by further fine-tuning the microcavity temperature and the radio frequency added to the acousto-optic modulator.

By setting the frequency interval of the two pump lasers to about one-half of the microcavity’s free spectral range, the comb teeth are staggered (approximately equally spaced distribution) to ensure that each comb tooth of the two SMCs can be clearly distinguished from the measured spectra when only one-shot spectral sampling is performed, thus eliminating the AME. The spectra of this two SMCs are shown in Fig. 2B, with their repetition rates (Fig. 2C) both around 49 GHz and adjustable by approximately 30 MHz. For a ranging system, the combined CDMC beam is transmitted to the free space through a collimator. The CDMC is then split into measuring and reference beams by a beam splitter and is recycled after being reflected by the target and reference mirror. The interference spectrum is measured using an optical spectrum analyzer (OSA), which carries the distance information.

### Distance measurement results

**Fig. 3.**
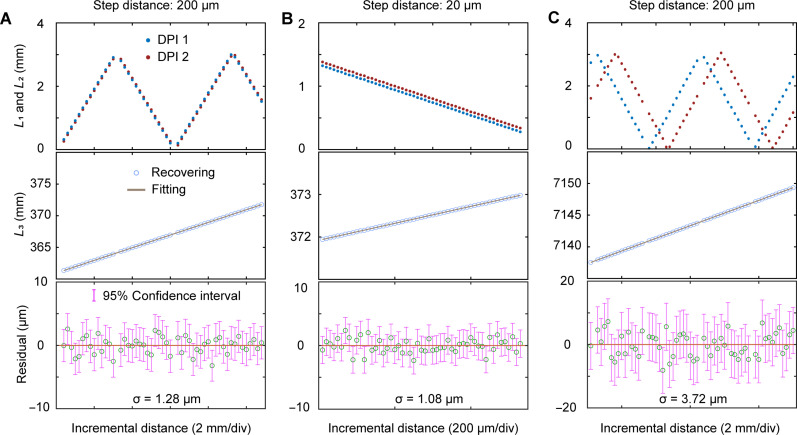
Experimental measurement results for different distances. (**A**) and (**B**) show the moving ranging results with step sizes of 200 and 20 μm, respectively, near a 370-mm round-trip distance, and (**C**) shows the moving ranging results with a 200-μm step size near a 7145-mm round-trip distance. Top, the aliased distances of two groups with the shape of triangular wave directly solved. Middle, the absolute distances recovered according to the Vernier effect. Bottom, the residuals of the increasing absolute distances and their linear fitting. The residuals are all less than 15 μm, and the SDs σ are 1.28, 1.08, and 3.72 μm, respectively.

We first validated the scheme’s ability to extend NAR by measuring different distances, and the results are shown in Fig. 3. The repetition-rate difference of the set CDMC is 7.7 MHz, and the corresponding NAR is about 19.4 m. Figure 3 (A and B) shows the measured results when the target distance is about 370 mm and the targets move with steps of 200 and 20 μm, respectively. The two groups of aliased distances $L_{1}$ and $L_{2}$ solved directly exhibit a “triangular wave” pattern with the increase in the real distance. To verify the long-distance ranging capacity, the target is moved to a distance of 7145 mm. The target is also moved forward at a step of 200 μm, and Fig. 3C shows the measured results. In the vicinity of aliased distance 0 and $l_{\mathrm{pp}1}/2$ , it is difficult to accurately calculate the flight delay time τ due to pulse overlap, which is a common problem in the OFCs-based ranging systems. This issue can be resolved by changing the repetition rates of the dual microcombs. In addition, a dispersive Fourier transform–based dual-comb ranging method is also proposed recently to solve this problem by using in-pulse interference information (*45*). The middle panels show the linear increase of the absolute distances L with the target moving steps. The bottom panels show the residuals of the increasing absolute distance and their linear fitting, along with the confidence intervals under 95% confidence level. It can be seen that the residuals are all less than 15 μm, and the SDs σ are 1.28, 1.08, and 3.72 μm, respectively. The error primarily originates from the mechanical error of the stepper motor and the vibration of the experimental environment.

**Fig. 4.**
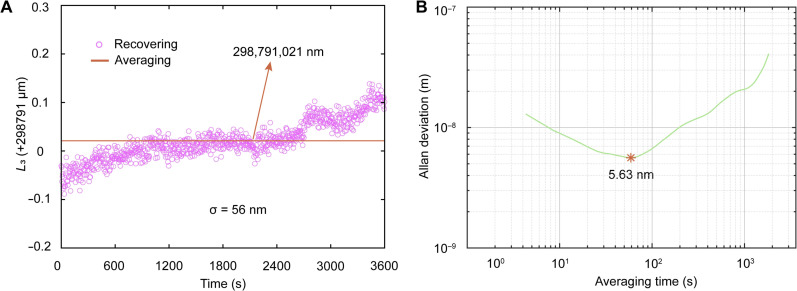
Fixed-point repeatability measurement results. (**A**) The results of multiple measurements within 1 hour with an absolute error of less than 300 nm and an SD of 56 nm. (**B**) The corresponding Allan deviation within 1 hour with a minimum value of 5.63 nm@56 s.

To avoid the influence of ambient vibration on the optical paths, we use an on-chip Mach-Zehnder interferometer (MZI; see fig. S2C) instead of the free-space interference optical path to evaluate the system performance more accurately. Figure 4A displays the results of continuous measurements over 1 hour, showing an optical path difference of 298,791 μm between the two arms of the MZI, with an absolute error of less than 300 nm and an SD of 56 nm. Figure 4B shows the corresponding Allan deviation of multiple measurements within 1 hour. When the averaging time increases to 56 s, the Allan deviation reaches a minimum value of 5.63 nm, which proves that the on-chip CDMC ranging system has high-accuracy ranging capability and robust stability. The Allan deviation deteriorates with the increase in the average time, which is mainly due to ambient temperature drift.

**Fig. 5.**
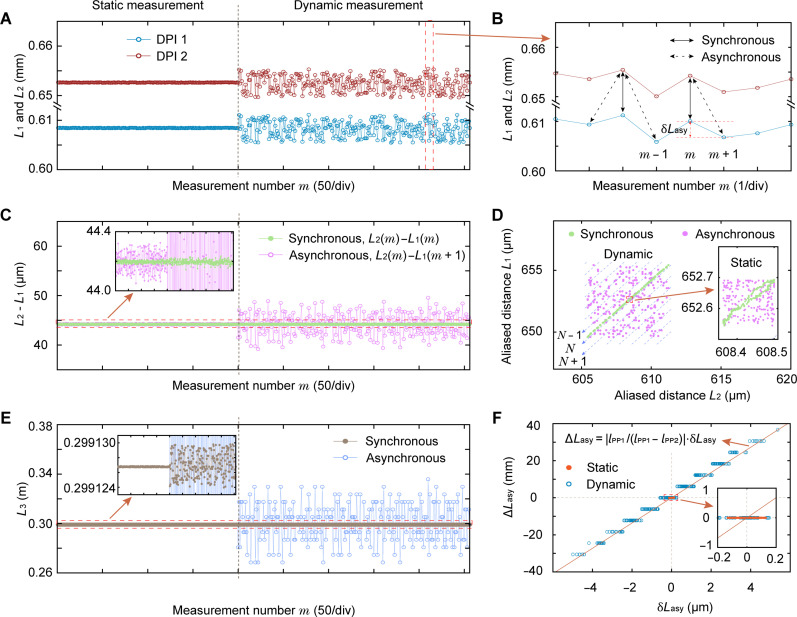
Comparative experimental results of synchronous and asynchronous measurements. (**A**) Jitter comparison of aliasing distances calculated under static and dynamic measurements. (**B**) Zoom-in of the dynamic measurement result, which illustrates the data combination settings for synchronous and asynchronous measurements. (**C**) and (**D**) show the $L_{2}-L_{1}$ values and the two-dimensional mapping of ( $L_{2}$ , $L_{1}$ ), respectively. (**E**) The recovered actual distance, where the results of the asynchronous measurement have a large error. (**F**) The corresponding relationship between $\delta L_{\mathrm{asy}}$ and $\Delta L_{\mathrm{asy}}$.

The advantage of the CDMC ranging scheme in eliminating AME is demonstrated by further comparison experiments involving synchronous and asynchronous measurement. We use another on-chip MZI to simulate static and dynamic ranging scenarios, where the optical path difference can be precisely controlled and tuned using a temperature controller. Although the scanning-based sampling of OSA used in the validation experiment is inherently asynchronous, the distance to be measured during each sampling can be fixed by temperature controller, thus ensuring the synchronization of the one-shot spectral sampling. As shown in Fig. 5A, for static measurement (where the temperature of the MZI is held constant at 25°C), the calculated $L_{1}$ and $L_{2}$ values jitter less than 200 nm. For dynamic measurement (where the temperature is randomly tuned within the range of 25° ± 1°C), the random jitter increases to about 6 μm. However, from the zoomed-in dynamic measurement result in Fig. 5B, it is clear that $L_{1}$ and $L_{2}$ values jitter at the same pace. This synchronous jitter implies that we can accurately calculate the actual distance, although it is changing dynamically. In addition, when the measurement numbers of $L_{1}$ and $L_{2}$ are consistent, it indicates synchronous measurement, and when they are inconsistent, it indicates asynchronous measurement. For example, [ $L_{2}\left(m\right)$ , $L_{1}\left(m\right)$ ] represents a set of synchronous measurement values, while [ $L_{2}\left(m\right)$ , $L_{1}\left(m+1\right)$ ] represents a set of asynchronous measurement values. The following comparative calculations are based on these synchronous and asynchronous measurement settings. Figure 5D shows the two-dimensional mapping of ( $L_{2}$ , $L_{1}$ ) values under synchronous and asynchronous measurement settings. For synchronous measurement, the [ $L_{2}\left(m\right)$ , $L_{1}\left(m\right)$ ] values approximately exhibit a linear distribution consistent with the theory, and it is easy judge from Fig. 1C that these values satisfy the corresponding cases of Eq. 2 (a or c). However, for asynchronous measurement, the [ $L_{2}\left(m\right)$ , $L_{1}\left(m+1\right)$ ] values are randomly distributed within a square region with a side length equal to the jitter range, leading to errors in the calculation of the integer N in Eq. 2 in the case of dynamic measurements. Since the integer N in Eq. 2 (a or c) has a linear relationship with the difference values $L_{2}-L_{1}$ , we give the $L_{2}-L_{1}$ values of synchronous and asynchronous measurements in Fig. 5C and find that the jitter of $L_{2}-L_{1}$ values of synchronous measurements is smaller than that of asynchronous measurements. The jitter of the $L_{2}-L_{1}$ values is maintained at less than 92 nm for both static and dynamic measurements, which means that the one-shot ranging resolution is 92 nm. Figure 5E shows the recovered actual distance, and there is little difference between the results of the asynchronous and synchronous measurement in the static case. In the dynamic case, the asynchronous measurement produces a huge error of about 60 mm in the actual distance due to the calculation error of the integer N , whereas this error is completely eliminated in the synchronous measurement. In addition, the corresponding relationship between $\delta L_{\mathrm{asy}}$ and $\Delta L_{\mathrm{asy}}$ calculated is entirely consistent with the theoretical error Eq. 3, where the step characteristic of the data is caused by the integer operation in the calculation of N (see Fig. 5F).

## DISCUSSION

From Eq. 1, it can be seen that the extended NAR is inversely proportional to the repetition-rate difference, i.e., a smaller repetition-rate difference corresponds to a larger NAR. By choosing microcavities with similar free-spectral range, the repetition-rate difference of the CDMC can be adjusted within the range of 0 to 60 MHz (see fig. S2B), which theoretically extends the NAR to an infinite length. However, considering the impact of single-comb ranging accuracy on NAR, the actual NAR is limited by repetition-rate instability, as well as other factors such as sampling error and algorithmic error, with repetition-rate instability being a major factor.

**Fig. 6.**
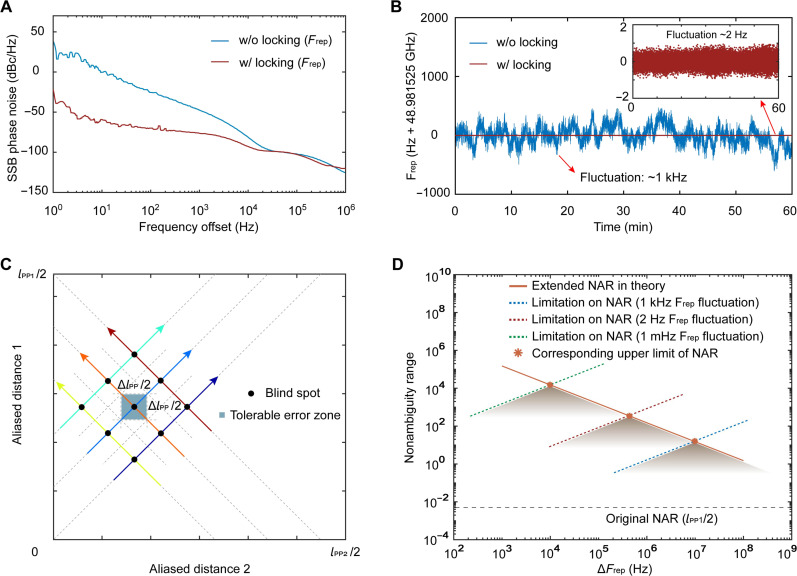
The NAR extension and error analysis. (**A**) The single-sideband phase noise comparison of the repetition-rate signals under conditions with locking and without locking. (**B**) The comparative results of long-term jitter in the repetition-rate. (**C**) Schematic diagram of blind spots and tolerable error zones. (**D**) The correspondence between the NAR and the repetition-rate difference (see Eq. 1), and the limitation of the repetition-rate jitter on the NAR (see Eq. 4), where the intersections of two lines correspond to the upper limits of NAR (see Eq. 5).

Next, the effects of repetition-rate jitter on measurement accuracy and NAR are analyzed when sampling and algorithm errors are ignored. The distance between two adjacent pulse is $l_{\mathrm{pp}}=c/f_{\mathrm{rep}}$ , and the pulse interval jitter caused by repetition-rate instability ${\delta f}_{\mathrm{rep}}$ is $\delta l_{\mathrm{pp}}=\left(c/{f_{\mathrm{rep}}}^{2}\right)\cdot {\delta f}_{\mathrm{rep}}$ . This leads to measurement errors in $L_{1}$ and $L_{2}$ so that the corresponding coordinate points may deviate from the oblique lines in Fig. 1C. To determine the corresponding trajectory of the measured coordinate points, the distance between these coordinate points and the parallel lines must be less than half the spacing between adjacent parallel lines (see Fig. 6C). It is also required that the errors in $L_{1}$ and $L_{2}$ are both less than $\Delta l_{\mathrm{pp}}/4$ , where $\Delta l_{\mathrm{pp}}=l_{\mathrm{pp}2}-l_{\mathrm{pp}1}=c\cdot ∆f_{\mathrm{rep}}/\left(f_{\mathrm{rep}1}\cdot f_{\mathrm{rep}2}\right)$ . The error in the aliasing distance $L_{i}\;\left(i=1,2\right)$ can be derived using the differential of Eq. 2 as $\delta L_{i}=N\cdot \delta l_{\mathrm{pp}}<\Delta l_{\mathrm{pp}}/4$ . Therefore, the actual NAR limited by repetition-rate instability is

$$L_{\mathrm{NAR}4}=N\cdot l_{\mathrm{pp}}=\Delta l_{\mathrm{pp}}\cdot l_{\mathrm{pp}}/\left(4\cdot \delta l_{\mathrm{pp}}\right)=c\cdot ∆f_{\mathrm{rep}}/\left(4f_{\mathrm{rep}}\cdot {\delta f}_{\mathrm{rep}}\right)$$ (4)

To maximize the extension of the NAR, we used the well-developed microwave injection locking method (see the “Supplementary Materials”) to improve the repetition-rate stability. Figure 6A shows the single-sideband phase noise comparison of the repetition-rate signals under locked and unlocked conditions, where the phase noise of the injection-locked repetition rate is notably reduced in the lower frequency region. Figure 6B shows the comparative results of repetition-rate fluctuations over 1 hour, demonstrating that the peak-to-peak jitter of the repetition-rate decrease from ~1 kHz to ~2 Hz with locking. Figure 6D illustrates the correspondence between the NAR and the repetition-rates difference in Eq. 1 and the restriction of repetition-rate stability on the NAR in Eq. 4. The NAR is concurrently constrained by both the repetition-rate difference and instability, where the intersection of two lines indicates the maximum NAR. According to Eqs. 1 and 4, let $L_{\mathrm{NAR}4}=L_{\mathrm{NAR}3}$ , and the NAR takes the upper limit value

$$L_{\mathrm{NAR}-\text{max}}=c/\left(2\sqrt{2f_{\mathrm{rep}}\cdot {\delta f}_{\mathrm{rep}}}\right)$$ (5)

where $∆{f_{\mathrm{rep}}}^{2}=2f_{\mathrm{rep}}\cdot {\delta f}_{\mathrm{rep}}$ . It can be concluded that the upper limit of the NAR corresponding to a 2-Hz repetition-rate instability is 339 m. To date, by locking the repetition rate to an atomic clock-referenced microwave signal (*42*, *46*), the repetition-rate instability of SMC has been reduced to sub-millihertz, which can raise the upper limit of NAR to over 10 km, ensuring the feasibility of the proposed scheme for long-distance ranging. The ranging accuracy based on DPI method is theoretically unrelated to the absolute frequency stability of the comb teeth. However, if the frequency of the pump laser can be locked, the absolute frequency interference information of the comb tooth can be used to further improve the ranging accuracy. The frequency of SMC can be fully locked through the technical solution of f-2f (*47*) or 2f-3f (*48*). If implemented, the NAR and accuracy of the CDMC ranging system will no longer be limited by frequency jitter and have the ability to measure distances over hundred kilometers.

In addition, as shown in Fig. 6C, the intersections of all oblique lines are theoretically a series of measurement blind spots. For example, substituting the corresponding $L_{1}$ and $L_{2}$ values from the intersection point into Eq. 2, results in two absolute distances that satisfy the decision conditions. These two distances belong to the two oblique lines passing through the intersection point, respectively, causing ambiguity in distance. In practice, we can confirm the moving direction of $L_{1}$ and $L_{2}$ coordinate points by fine-tuning the repetition rate or the length of the reference path and then identify which line the intersection points belong to, thus eliminating the ambiguity.

In the proof-of-principle experiment, only a slow-scanning OSA was used for sampling, which limited the potential measurement speed of the CDMC system. If a commercial high-speed spectrometer is adopted, a measurement speed of 10 kHz could be achieved, while using a PD array could enable higher speeds. Although the CDMC ranging method relies on spectral measurements, it ultimately calculates distance through temporal pulse delay information. Therefore, wavelength-dependent losses (e.g., the presence of absorbing substances in the ranging path) and the nonsmoothness of the SMCs spectral envelopes themselves exhibit negligible effects on measurement accuracy.

Furthermore, thanks to the rapid development of photonic integrated chips, every component in this ranging system can be easily integrated onto a CMOS-compatible material platform. Currently, SMC generation systems containing semiconductor lasers and microring resonators have achieved monolithic heterogeneous integration (*49*), and high-performance on-chip optical amplifiers (*50*), transceiver antennas (*51*), and array waveguide gratings (*52*) have also been demonstrated. To achieve fast one-to-one synchronous detection of all combs on chip, PD arrays are key devices, and fortunately, the on-chip demonstration of highly sensitive APDs (*53*) and large-scale balanced detectors (*54*) makes this possible. Analog-to-digital converters for real-time acquisition of the large number of electrical signals generated by PDs, and FPGAs or application-specific integrated circuits for further digital signal processing can also be integrated with other components using photonic wire-bonding technology (*55*, *56*).

In summary, we propose an on-chip CDMC ranging scheme that leverages the Vernier effect generated by two SMCs to extend the NAR, and it requires only a single DPI spectrum measurement to determine the distance. The modes of the dual microcombs intersect regularly, facilitating a nearly equidistant distribution, which greatly benefits the one-to-one mapping with the linear array detector. This simplifies spectral measurements and reduces data volume, thus promoting the integration and miniaturization of the overall system. At a round-trip distance of 7.145 m, the SD of stepwise measurements is 3.72 μm. Long-term stable precision measurement is also demonstrated at 0.3 m with a minimum Allan deviation of 5.63 nm at an averaging time of 56 s. In addition, the advantage on AME elimination is demonstrated by further comparison experiments. We also analyze the influence of repetition-rate instability on NAR, revealing an inverse square relationship between the upper limit of NAR and repetition-rate instability. The NAR of proposed SDMC ranging scheme is extended to 339 m by locking the repetition rate of SMCs, and has the potential to extend to tens of kilometers or more. Therefore, the proposed scheme may find important application in long-distance measurements.

## MATERIALS AND METHODS

### Data processing process of CDMC ranging

**Fig. 7.**
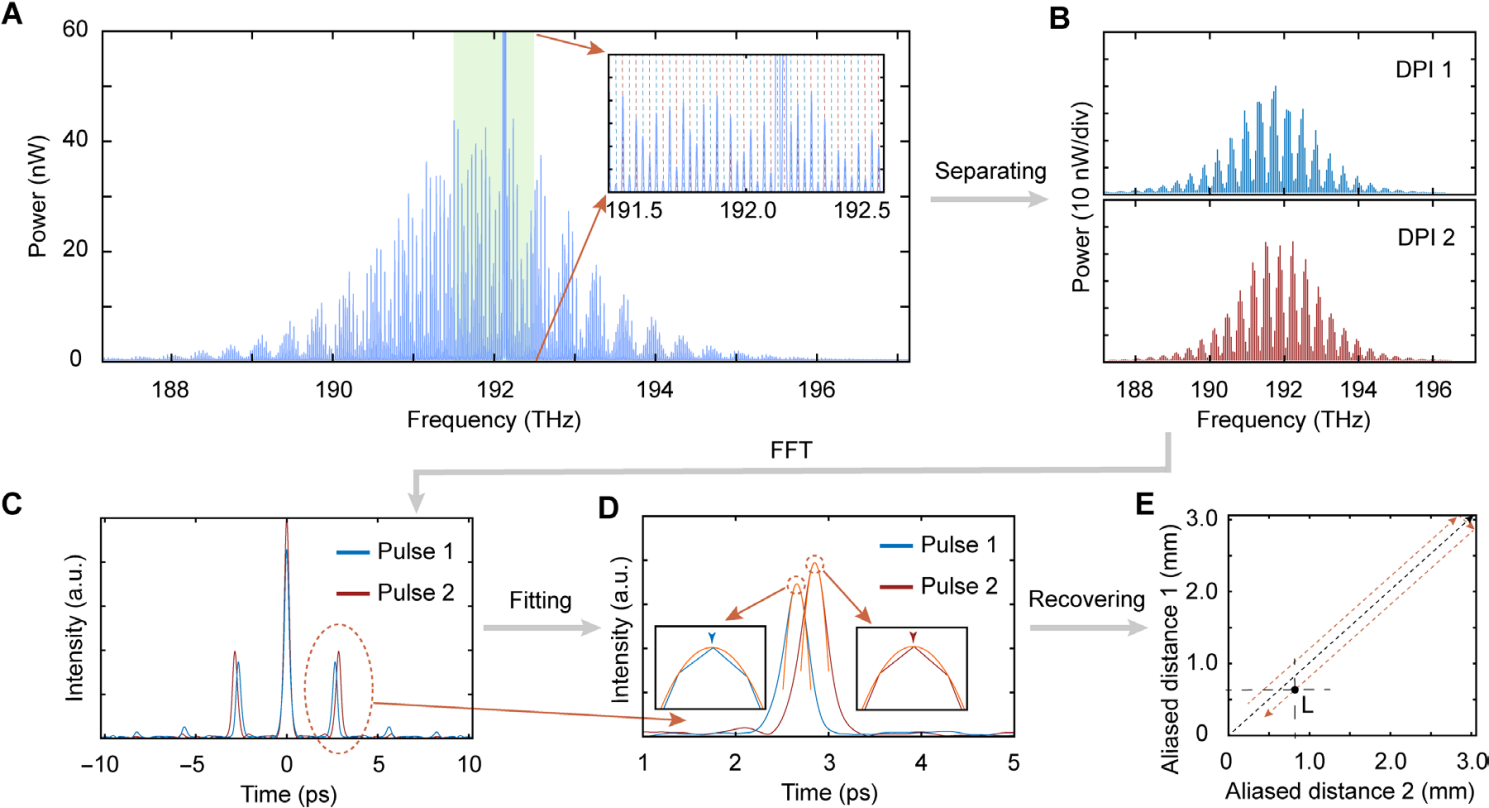
Data processing process of the CDMC ranging. (**A**) A DPI spectrum of on-chip CDMC containing distance information. (**B**) Two DPI spectra after separating. (**C**) Pseudo-time domain information obtained after FFT. (**D**) The peaks value of the pseudo-time domain pulse envelope is obtained by polynomial fitting. (**E**) Two aliasing distances are obtained, and absolute distances are recovered according to the Vernier effect. a.u., arbitrary units.

The data processing process of the CDMC ranging system as shown in Fig. 7. Firstly, the DPI spectra in Fig. 7A are separated after filtering out the strong pumping components (see Fig. 7B). The frequency coordinates of DPI spectra are redefined according to the pumping frequencies and repetition rates. Then, the pseudo-time domain information in Fig. 7C is obtained by performing a fast Fourier transform (FFT) on the DPI spectra, where two weaker impulse functions are symmetrically distributed on either side of a stronger impulse function. The peak values of the FFT signals are obtained by polynomial fitting in Fig. 7D, which denote the transmission time τ of the under-test distance. Two aliasing distances $L_{1}$ and $L_{2}$ can be calculated according to formula L=cτ/n . Last, the real absolute distance L is obtained according to the distance recovery Eq. 2 (see Fig. 7E). The ambient temperature, humidity and atmospheric pressure monitored during the experiment were 22.9°C, 40.8%, and 99,460 Pa, respectively. According to Edllén formula (*57*), the refractive index n of air could be calculated as 1.0002606. The same repetition-rate values are applied when processing multi-measurement data. By synchronously monitoring the instantaneous repetition rates during the measurement of DPI spectra, the repetition rates can be corrected during data processing, thereby improving ranging accuracy.

## Supplementary Materials

### This PDF file includes:

Supplementary Text Figs. S1 to S3 Table S1 References
