# Supplementary material for: Cross dual-microcomb dispersion interferometry ranging
Source: Sci Adv. 2025 Aug 15;11(33):eadt4252. doi: 10.1126/sciadv.adt4252 (PMC12356267; doi:10.1126/sciadv.adt4252)
Supplement: Supplementary file 1 — Supplementary Text Figs. S1 to S3 Table S1 References [file sciadv.adt4252_sm.pdf]

Science Advances
AAAS

Supplementary Materials for

Cross dual-microcomb dispersion interferometry ranging

Yang Wang *et al.*

Corresponding author: Wenfu Zhang, wfuzhang@opt.ac.cn

Sci. Adv. **11**, eadt4252 (2025)
DOI: 10.1126/sciadv.adt4252

This PDF file includes:

Supplementary Text
Figs. S1 to S3
Table S1
References

Supplementary Text

Supplementary Note S1. The SMC spectral characteristics and ranging experimental settings

As shown in **Fig. S1**, the SMC's power used in the ranging experiments is about 1~2 mW (0~3 dBm, excluding the strong pump light), and the number of comb-teeth used is about 360 (from 183 to 201 THz). The SMCs do not require power amplification and waveform shaping, and can be directly connected to the ranging optical path for measurement with 9-port light after 1:9 beam splitting. The received interference spectrum power depends on the optical coupling efficiency of the ranging system, which is usually between 1~500 uW. The spectrometer used in the experiment is the YOKOGAWA-AQ6370B, which has a minimum resolution of 0.02 nm (~2.5 GHz). In order to clearly distinguish the comb-teeth of CDMC, we set the resolution to 0.02 nm, corresponding to a single frame acquisition speed of about 1 s, with an additional 2~3 s for data storage.

Supplementary Note S2. Extended measurements

We use an on-chip Mach-Zehnder interferometer (MZI, see **Fig. S2C**) instead of the free-space interference optical path to evaluate the system performance under different repetition-rate differences. As shown in **Fig. S2A**, the repetition-rate of the SMC can be tuned about 30 MHz through the thermo-optic effect with a tuning efficiency of 0.43 MHz/°C. **Figure S2B** shows the variation of the repetition-rate of single SMCs based on 7 different microcavities on the same wafer through tuning the operating temperature. The similarity and tunability of the repetition-rate ensure that the repetition-rate difference can be continuously adjusted within the range of 0 to 60 MHz. **Figure S2D** shows the ranging results under different repetition-rate differences. The top panels show the two groups of aliased distances directly solved under the repetition-rate difference of 1.65, 5.20, 7.30, 9.05, and 11.20 MHz, while the bottom panels show the corresponding absolute distances recovered according to the Vernier effect. The measured results under these five different repetition-rate differences are consistent, confirming that the optical path difference of the on chip MZI is about 298791 um. The standard deviations are 50, 58, 74, 38, and 33 nm respectively, and this variation is due to the algorithmic errors and the limited number of measurements.

Supplementary Note S3. Experimental results concerning the injection locking of the repetition-rate

When generating SMCs using the laser-assisted intracavity thermal balance scheme, we utilized an acousto-optic modulator (AOM) to naturally lock the beat frequency of the pump and auxiliary lasers, stabilizing the repetition-rate jitter at approximately 1 kHz. This limited the NAR of the ranging to just over ten meters. To further extend the NAR, we employed RF injection locking technology (*42, 46, 58, 59*) to reduce the jitter of the repetition-rates (F_{rep}) and its difference (ΔF_{rep}). **Figure S3A** provides a schematic diagram of the experimental setup for the injection locking scheme, which differs slightly from the previous SMC generation experimental setup by introducing intensity modulator (IM) to the pump light before it enters the EDFA. When the modulation sidebands induced by the RF signal match the repetition-rate of the SMC, the repetition-rate can be locked. **Figure S3B** shows the single-sideband (SSB) phase noise comparison results of the F_{rep} and ΔF_{rep} before and after locking, as measured by a phase noise analyzer (PNA, ROHDE&SCHWARZ-FSWP50). It is evident that after injection locking, the phase noise of the F_{rep} and ΔF_{rep} notablely decreases at lower frequencies. **Figures S3C** and **S3F** present the linewidth comparison results of the F_{rep} and ΔF_{rep} before and after locking, respectively. **Figures S3D** and **S3G** show respectively the long-term jitter comparison results of the F_{rep} and ΔF_{rep} before and after locking, as measured by a frequency counter (KEYSIGHT 53230A). After locking, the jitter of the F_{rep} and ΔF_{rep} decreased from approximately 1 kHz to about 2 Hz and 3 Hz, respectively. Based on the formulas provided in the main text, the corresponding NAR was calculated to be 339 m. **Figures S3E** and **S3H** present the relative Allan deviation of the F_{rep} and ΔF_{rep} jitter before and after locking, respectively.

Supplementary Note S4. Performance comparisons

In order to comprehensively evaluate the performance of the proposed CDMC ranging scheme in this work, the ranging accuracy, speed, resolution, non-ambiguity range (NAR) and asynchronous measurement error (AME) of the scheme are compared with those of other dual-comb ranging works. As shown in **Table S1**, the proposed scheme achieves relatively higher ranging accuracy but requires longer averaging time due to the slow sampling rate of the OSA used in our work. If high-speed spectrometer (such as: I-MON 512 HIGH SPEED USB3) or PD arrays are used in the future, it is expected to achieve nanometer-level accuracy in averaging times on the order of milliseconds. For the measurement speed, the time-domain asynchronous sampling

method is used in other works, so the sampling speed is equal to the repetition-rate difference. However, resolving absolute distances usually requires secondary sampling (e.g., exchanging the dual-comb roles), which inherently limits their practical ranging speeds. Regarding measurement resolution, most prior works adopt the time-of-flight method in time domain, resulting in relatively low resolution of one-shot measurement. Notably, the resolution values in many works are worse than their accuracy values because accuracy is calculated using Allan deviation with multiple averaging, whereas resolution reflects one-shot measurement performance. For NAR, *Ref.* (*12*) claims a 30 km NAR based solely on ΔF_{rep} (Eq. (1) in the main text) but neglects the limitations imposed by repetition-rate jitter (Eq. (4) in the main text). Regarding AME, *Ref.* (*18*) and *Ref.* (*38*) do not utilize the Vernier effect for NAR extension and thus do not introduce AME. In contrast, *Ref.* (*12*), *Ref.* (*17*), *Ref.* (*37*), and *Ref.* (*44*) use the Vernier effect to extend NAR, but do not consider AME. Overall, the proposed CDMC scheme demonstrates good performance across these metrics.

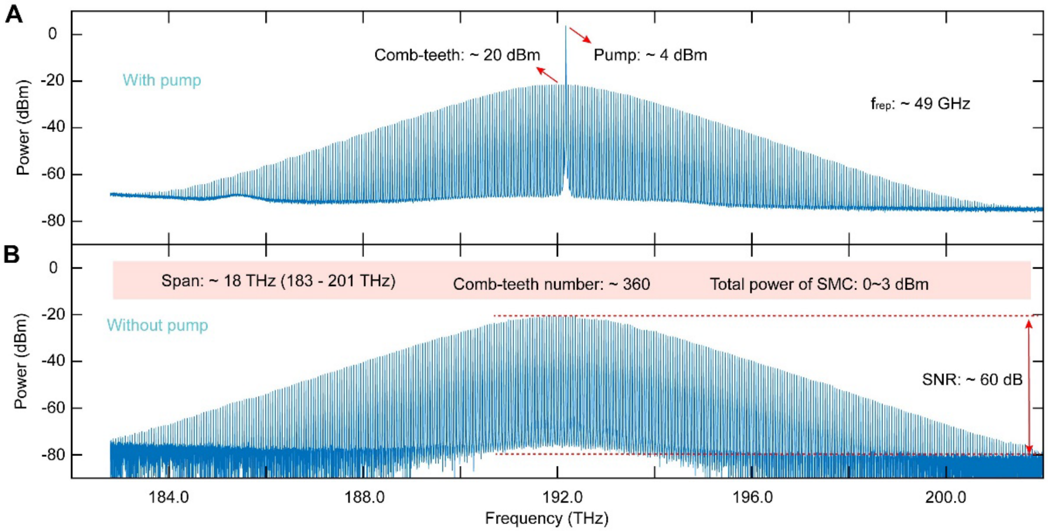

Fig. S1. Spectrum width and power of SMC. (A) With pump. (B) Without pump (SNR: signal-to-noise ratio).

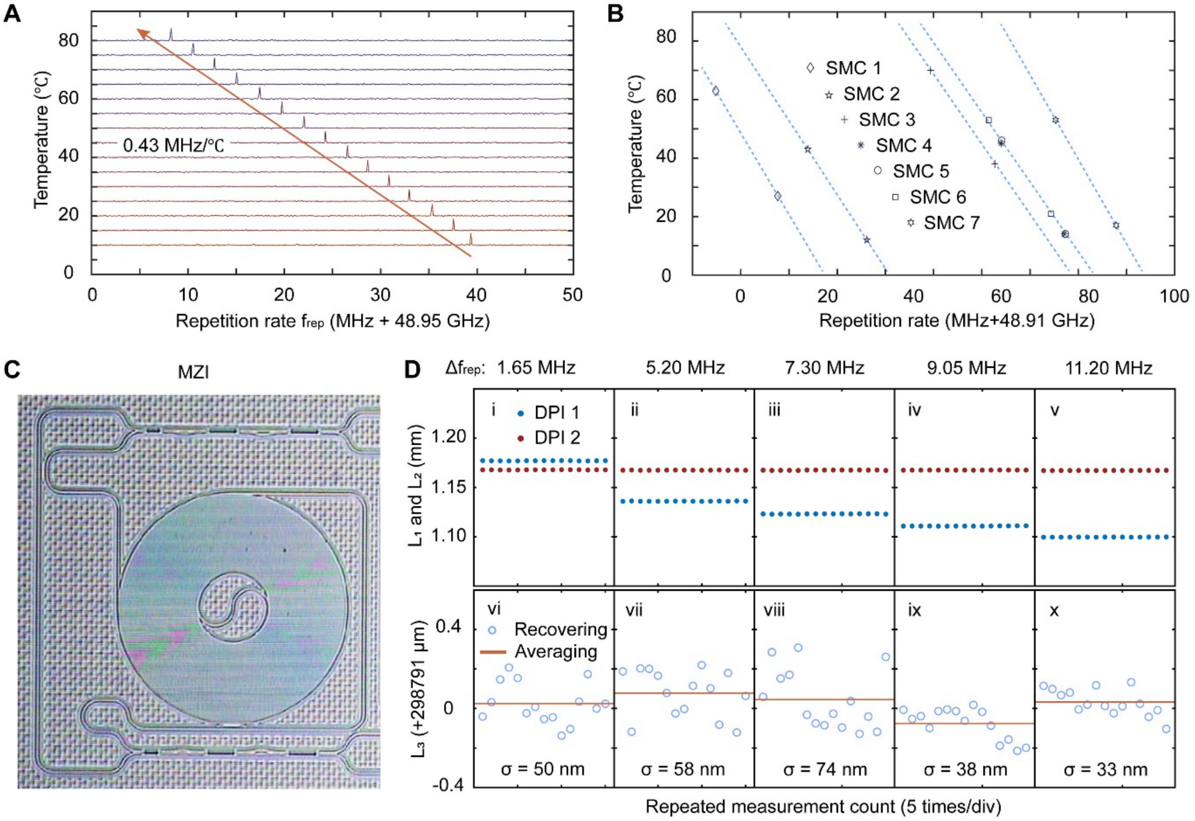

Fig. S2. Experimental measurement results under different repetition-rate differences. (A) The relationship between the repetition-rate and the temperature, and the tuning factor is about 0.43 MHz/°C. (B) The repetition-rates of several different single SMCs and their relationship with temperature. (C) On-chip Mach-Zehnder interferometer (MZI). (D) Fixed-point multiple ranging results under repetition-rate differences of 1.65, 5.20, 7.30, 9.05, and 11.20 MHz respectively, where the top panels show the aliasing distance and the bottom panels show the corresponding absolute distance and standard deviation.

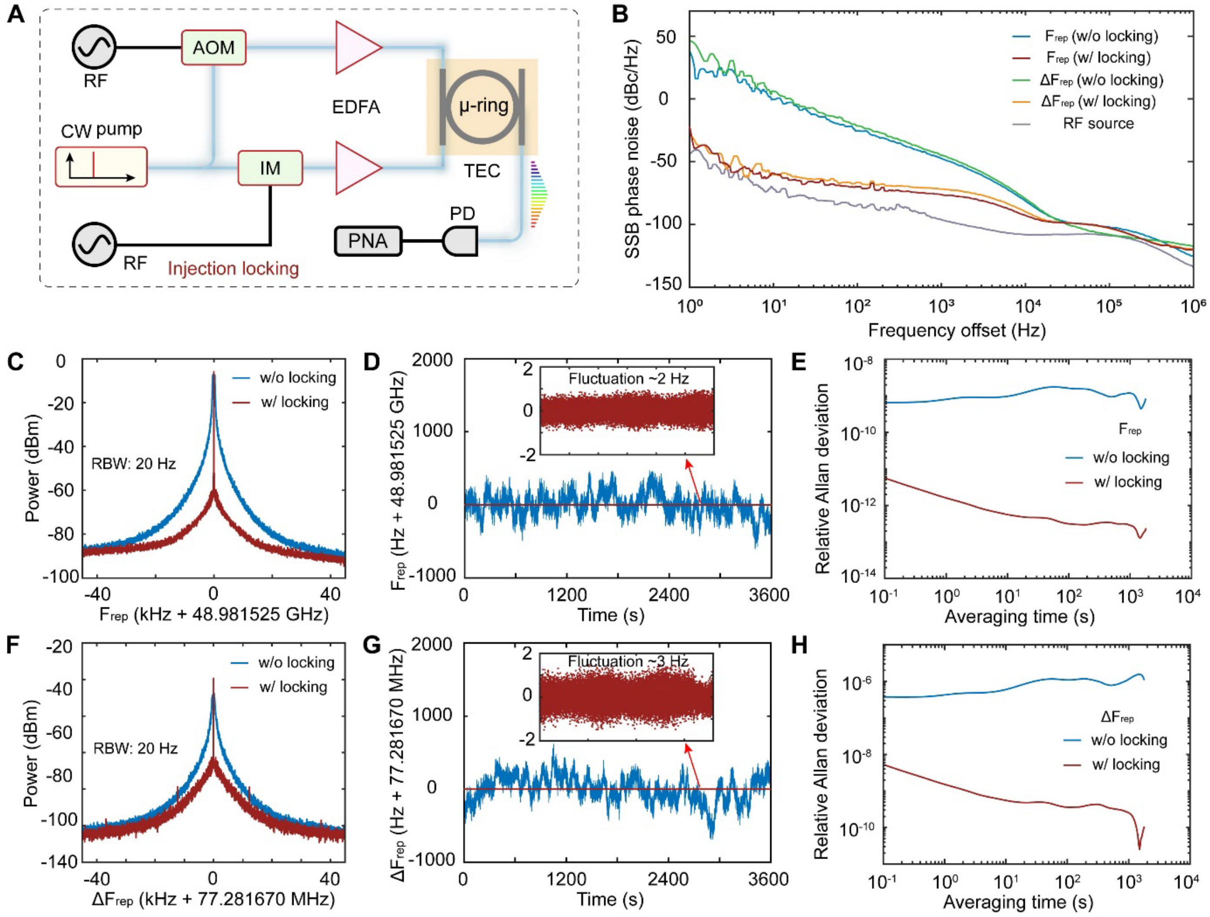

Fig. S3. Experimental results concerning the injection locking of the repetition-rate. (A) Experimental setup. (B) Single-sideband (SSB) phase noise. (C) and (F) are the linewidth comparison results of the $\mathbf{F_{rep}}$ and $\Delta\mathbf{F_{rep}}$ respectively. (D) and (G) are long-term jitter comparison results of the $\mathbf{F_{rep}}$ and $\Delta\mathbf{F_{rep}}$ respectively. (E) and (H) are the relative Allan deviation of the $\mathbf{F_{rep}}$ and $\Delta\mathbf{F_{rep}}$ respectively.

Table S1. Ranging performance comparison.

Ref	Accuracy (Allan deviation)	Speed **	Resolution (One-shot)	NAR	AME
(*12*)	3 nm@ 0.5 s	5 kHz (Matching with Δf_{rep})	~100 nm	30 km (f_{rep} jitter unaccounted)	Not eliminate
(*17*)	126 nm@ 1 s	5 kHz (Matching with Δf_{rep})	~100 um (Estimating)	1.26 km	Not eliminate
(*18*)	82.9 nm@ 500 ms	2 kHz (Matching with Δf_{rep})	~175 nm (Estimating)	693.7 mm (Not extended)	Do not exist
(*37*)	0.466 m@ 2 s	5.685 kHz (Matching with Δf_{rep})	~100 um (Estimating)	~26 km	Not eliminate
(*38*)	12 nm@ 13 us	96.4 MHz (Matching with Δf_{rep})	~ 1um (Estimating)	1.56 mm (Not extended)	Do not exist
(*44*)	0.71um (standard deviation)	few kHz (Matching with Δf_{rep})	~34 um	<112 mm (Estimating)	Not eliminate
**This work**	**5.63 nm@ 56 s ***	**Hz ~ MHz (Depending on the spectrometer sampling rate)**	**92 nm**	**339 m**	**Eliminate**

* At repetition rates of ~100 GHz and spectrometer sampling of 10 kHz (such as: I-MON 512 HIGH SPEED USB3), the required averaging time reaches the millisecond level.
** Other works reported single sampling speed (resolving fractional distances), while this work gave dual-synchronized sampling speed (resolving absolute distances).

REFERENCES AND NOTES

1. R. H. Schmitt, M. Peterek, E. Morse, W. Knapp, M. Galetto, F. Härtig, G. Goch, B. Hughes, A. Forbes, W. T. Estler, Advances in large-scale metrology-review and future trends. *CIRP Ann. Manuf. Technol.* **65**, 643–665 (2016).

2. S.-W. Kim, Metrology: Combs rule. *Nat. Photonics* **3**, 313–314 (2009).

3. F. Riehle, P. Gill, F. Arias, L. Robertsson, The CIPM list of recommended frequency standard values: Guidelines and procedures. *Metrologia* **55**, 188–200 (2018).

4. D. J. Jones, S. A. Diddams, J. K. Ranka, A. Stentz, R. S. Windeler, J. L. Hall, S. T. Cundiff, Carrier-envelope phase control of femtosecond mode-locked lasers and direct optical frequency synthesis. *Science* **288**, 635–639 (2000).

5. Z. L. Newman, V. Maurice, T. Drake, J. R. Stone, T. C. Briles, D. T. Spencer, C. Fredrick, Q. Li, D. Westly, B. R. Ilic, B. Q. Shen, M. G. Suh, K. Y. Yang, C. Johnson, D. M. S. Johnson, L. Hollberg, K. J. Vahala, K. Srinivasan, S. A. Diddams, J. Kitching, S. B. Papp, M. T. Hummon, Architecture for the photonic integration of an optical atomic clock. *Optica* **6**, 680–685 (2019).

6. X. Zheng, J. Dolde, V. Lochab, B. N. Merriman, H. R. Li, S. Kolkowitz, Differential clock comparisons with a multiplexed optical lattice clock. *Nature* **602**, 425–430 (2022).

7. K. Y. Wu, N. P. O'Malley, S. Fatema, C. Wang, M. Girardi, M. S. Alshaykh, Z. C. Ye, D. E. Leaird, M. H. Qi, V. T. Company, A. M. Weiner, Vernier microcombs for integrated optical atomic clocks. *Nat. Photonics* **19**, 400–406 (2025).

8. K. Minoshima, H. Matsumoto, High-accuracy measurement of 240 m distance in an optical tunnel by use of a compact femtosecond laser. *Appl. Opt.* **39**, 5512–5517 (2000).

9. J. Ye, Absolute measurement of a long, arbitrary distance to less than an optical fringe. *Opt. Lett.* **29**, 1153–1155 (2004).

10. K. N. Joo, S. W. Kim, Absolute distance measurement by dispersive interferometry using a femtosecond pulse laser. *Opt. Express* **14**, 5954–5960 (2006).

11. J. Jin, Y. J. Kim, Y. Kim, S. W. Kim, Absolute length calibration of gauge blocks using optical comb of a femtosecond pulse laser. *Opt. Express* **14**, 5968–5974 (2006).

12. I. Coddington, W. C. Swann, L. Nenadovic, N. R. Newbury, Rapid and precise absolute distance measurements at long range. *Nat. Photonics* **3**, 351–356 (2009).

13. J. Lee, Y. J. Kim, K. Lee, S. Lee, S. W. Kim, Time-of-flight measurement with femtosecond light pulses. *Nat. Photonics* **4**, 716–720 (2010).

14. S. A. van den Berg, S. T. Persijn, G. J. P. Kok, M. G. Zeitouny, N. Bhattacharya, Many-wavelength interferometry with thousands of lasers for absolute distance measurement. *Phys. Rev. Lett.* **10**, 183901 (2012).

15. E. Baumann, F. R. Giorgetta, I. Coddington, L. C. Sinclair, K. Knabe, W. C. Swann, N. R. Newbury, Comb-calibrated frequency-modulated continuous-wave ladar for absolute distance measurements. *Opt. Lett.* **38**, 2026–2028 (2013).

16. G. H. Wu, M. Takahashi, K. Arai, H. Inaba, K. Minoshima, Extremely high-accuracy correction of air refractive index using two-colour optical frequency combs. *Sci. Rep.* **3**, 1894 (2013).

17. J. Lee, S. Han, K. Lee, E. Bae, S. Kim, S. Lee, S. W. Kim, Y. J. Kim, Absolute distance measurement by dual-comb interferometry with adjustable synthetic wavelength. *Meas. Sci. Technol.* **24**, 045201 (2013).

18. H. Y. Zhang, H. Y. Wei, X. J. Wu, H. L. Yang, Y. Li, Absolute distance measurement by dual-comb nonlinear asynchronous optical sampling. *Opt. Express* **22**, 6597–6604 (2014).

19. H. Z. Wu, F. M. Zhang, T. Y. Liu, F. Meng, J. S. Li, X. H. Qu, Absolute distance measurement by chirped pulse interferometry using a femtosecond pulse laser. *Opt. Express* **23**, 31582–31593 (2015).

20. Z. B. Zhu, G. Y. Xu, K. Ni, Q. Zhou, G. H. Wu, Synthetic-wavelength-based dual-comb interferometry for fast and precise absolute distance measurement. *Opt. Express* **26**, 5747–5757 (2018).

21. Q. Vinckier, M. Tinto, I. Grudinin, D. Rieländer, N. Yu, Experimental demonstration of time-delay interferometry with optical frequency comb. *Phys. Rev. D* **102**, 062002 (2020).

22. T. Herr, V. Brasch, J. D. Jost, C. Y. Wang, N. M. Kondratiev, M. L. Gorodetsky, T. J. Kippenberg, Temporal solitons in optical microresonators. *Nat. Photonics* **8**, 145–152 (2014).

23. M. J. Yu, J. K. Jang, Y. Okawachi, A. G. Griffith, K. Luke, S. A. Miller, X. C. Ji, M. Lipson, A. L. Gaeta, Breather soliton dynamics in microresonators. *Nat. Commun.* **8**, 14569 (2017).

24. Q. F. Yang, B. Q. Shen, H. M. Wang, M. Tran, Z. W. Zhang, K. Y. Yang, L. Wu, C. Y. Bao, J. Bowers, A. Yariv, K. V. Q. F. Yang, B. Q. Shen, H. M. Wang, M. Tran, Z. W. Zhang, K. Y. Yang, L. Wu, C. Y. Bao, J. Bowers, A. Yariv, K. Vahala, Vernier spectrometer using couterpropagating soliton microcombs. *Science* **363**, 965–968 (2019).

25. H. Zhou, Y. Geng, W. W. Cui, S. W. Huang, Q. Zhou, K. Qiu, C. W. Wong, Soliton bursts and deterministic dissipative Kerr soliton generation in auxiliary-assisted microcavities. *Light Sci. Appl.* **8**, 50 (2019).

26. W. Q. Wang, L. R. Wang, W. F. Zhang, Advances in soliton microcomb generation. *Adv. Photon.* **2**, 034001 (2020).

27. H. M. Wang, Y. K. Lu, L. Wu, D. Y. Oh, B. Q. Shen, S. H. Lee, K. Vahala, Dirac solitons in optical microresonators. *Light Sci. Appl.* **9**, 205 (2020).

28. A. Tikan, A. Tusnin, J. Riemensberger, M. Churaev, X. R. Ji, K. N. Komagata, R. N. Wang, J. Q. Liu, T. J. Kippenberg, Protected generation of dissipative Kerr solitons in supermodes of coupled optical microresonators. *Sci. Adv.* **8**, eabm6982 (2022).

29. L. Chang, S. T. Liu, J. E. Bowers, Integrated optical frequency comb technologies. *Nat. Photon.* **16**, 95–108 (2022).

30. J. Liu, F. Bo, L. Chang, C. H. Dong, X. Ou, B. Regan, X. Q Shen, Q. H. Song, B. C. Yao, W. F. Zhang, C. L. Zou, Y. F. Xiao, Emerging material platforms for integrated microcavity photonics. *Sci. China Phys. Mech. Astron.* **65**, 104201 (2022).

31. Y. Wang, W. Q. Wang, Z. Z. Lu, X. Y. Wang, L. Huang, B. E. Little, S. T. Chu, W. Zhao, W. F. Zhang, Hyperbolic resonant radiation of concomitant microcombs induced by cross-phase modulation. *Photonics Res.* **11**, 1075–1084 (2023).

32. Y. Wang, Z. C. Wang, X. Y. Wang, W. Shao, L. Huang, B. Liang, B. E. Little, S. T. Chu, W. Zhao, W. Q. Wang, W. F. Zhang, Scanning dual-microcomb spectroscopy. *Sci. China Phys. Mech. Astron.* **65**, 294211 (2022).

33. Z. Y. Sun, Y. Li, B. F. Bai, Z. D. Zhu, H. B. Sun, Silicon nitride-based Kerr frequency combs and applications in metrology. *Adv. Photon.* **4**, 064001 (2023).

34. J. D. Wang, Z. Z. Lu, W. Q. Wang, F. M. Zhang, J. W. Chen, Y. Wang, J. H. Zheng, S. T. Chu, W. Zhao, B. E. Little, X. H. Qu, W. F. Zhang, Long-distance ranging with high precision using a soliton microcomb. *Photonics Res.* **8**, 1964–1972 (2020).

35. Y. S. Jang, H. Liu, J. H. Yang, M. B. Yu, D. L. Kwong, C. W. Wong, Nanometric precision distance metrology via hybrid spectrally resolved and homodyne interferometry in a single soliton frequency microcomb. *Phys. Rev. Lett.* **126**, 023903 (2021).

36. J. T. Li, B. Chang, J. T. Du, T. Tan, Y. Geng, H. Zhou, Y. P. Liang, H. Zhang, G. F. Yan, L. M. Ma, Z. L. Ran, Z. N. Wang, B. C. Yao, Y. J. Rao, Coherently parallel fiber-optic distributed acoustic sensing using dual Kerr soliton microcombs. *Sci. Adv.* **10**, eadf8666 (2024).

37. M. G. Suh, K. J. Vahala, Soliton microcomb range measurement. *Science* **359**, 884–887 (2018).

38. P. Trocha, M. Karpov, D. Ganin, M. H. P. Pfeiffer, A. Kordts, S. Wolf, J. Krockenberger, P. Marin-Palomo, C. Weimann, S. Randel, W. Freude, T. J. Kippenberg, C. Koos, Ultrafast optical ranging using microresonator soliton frequency combs. *Science* **359**, 887–891 (2018).

39. J. Riemensberger, A. Lukashchuk, M. Karpov, W. Weng, E. Lucas, J. Q. Liu, T. J. Kippenberg, Massively parallel coherent laser ranging using soliton microcombs. *Nature* **581**, 164–170 (2020).

40. L. H. Jia, Y. Wang, X. Y. Wang, F. M. Zhang, W. Q. Wang, J. D. Wang, J. H. Zheng, J. W. Chen, M. Y. Song, X. Ma, M. Y. Yuan, B. Little, S. T. Chu, D. Cheng, X. H. Qu, W. Zhao, W. F. Zhang, Nonlinear calibration of frequency modulated continuous wave LIDAR based on a microresonator soliton comb. *Opt. Lett.* **46**, 1025–1028 (2021).

41. R. X. Chen, H. W. Shu, B. T. Shen, L. Chang, W. Q. Xie, W. C. Liao, Z. H. Tao, J. E. Bowers, X. J. Wang, Breaking the temporal and frequency congestion of LiDAR by parallel chaos. *Nat. Photon.* **17**, 306–314 (2023).

42. M. Y. Yang, G. C. Wang, Z. C. Wang, X. B. Li, L. X. Zhu, W. Q. Wang, W. F. Zhang, S. H. Yan, J. Yang, Micrometer-precision absolute distance measurement with a repetition-rate-locked soliton microcomb. *Opt. Lett.* **48**, 4356–4359 (2023).

43. D. R. Carlson, D. D. Hickstein, D. C. Cole, S. A. Diddams, S. B. Papp, Dual-comb interferometry via repetition rate switching of a single frequency comb. *Opt. Lett.* **43**, 3614–3617 (2018).

44. S. Bak, G. H. Kim, H. Jang, C. S. Kim, Optical Vernier sampling using a dual-comb-swept laser to solve distance aliasing. *Photonics Res.* **9**, 657–667 (2021).

45. B. Chang, T. Tan, J. T. Du, X. Y. He, Y. P. Liang, Z. R. Liu, C. Wang, H. D. Xia, Z. H. Wu, J. D. Wang, K. Y. Wong, T. Zhu, L. J. Kong, B. W. Li, Y. J. Rao, B. C. Yao, Dispersive Fourier transform based dual-comb ranging. *Nat. Commun.* **15**, 4990 (2024).

46. R. Niu, S. Wan, T. P. Hua, W. Q. Wang, Z. Y. Wang, J. Li, Z. B. Wang, M. Li, Z. Shen, Y. R. Sun, S. M. Hu, B. E. Little, S. T. Chu, W. Zhao, G. C. Guo, C. L. Zou, Y. F. Xiao, W. F. Zhang, C. H. Dong, Atom-referenced and stabilized soliton microcomb. *Sci. China Phys. Mech. Astron.* **67**, 224262 (2024).

47. P. Del'Haye, A. Coillet, T. Fortier, K. Beha, D. C. Cole, K. Y. Yang, H. Lee, K. J. Vahala, S. B. Papp, S. A. Diddams, Phase-coherent microwave-to-optical link with a self-referenced microcomb. *Nat. Photon.* **10**, 516–520 (2016).

48. V. Brasch, E. Lucas, J. D. Jost, M. Geiselmann, T. J. Kippenberg, Self-referenced photonic chip soliton Kerr frequency comb. *Light Sci. Appl.* **6**, e16202 (2017).

49. C. Xiang, J. Q. Liu, J. Guo, L. Chang, R. N. Wang, W. Weng, J. Peters, W. Q. Xie, Z. Y. Zhang, J. Riemensberger, J. Selvidge, T. J. Kippenberg, J. E. Bowers, Laser soliton microcombs heterogeneously integrated on silicon. *Science* **373**, 99–103 (2021).

50. Y. Liu, Z. R. Qiu, X. R. Ji, A. Lukashchuk, J. J. He, J. Riemensberger, M. Hafermann, R. N. Wang, J. Q. Liu, C. Ronning, T. J. Kippenberg, A photonic integrated circuit–based erbium-doped amplifier. *Science* **376**, 1309–1313 (2022).

51. S. A. Miller, Y. C. Chang, C. T. Phare, M. C. Shin, M. Zadka, S. P. Roberts, B. Stern, X. C. Ji, A. Mohanty, O. A. Jimenez Gordillo, U. D. Dave, M. Lipson, Large-scale optical phased array using a low-power multi-pass silicon photonic platform. *Optica* **7**, 3–6 (2020).

52. S. Y. Ren, W. Q. Wang, Y. J. Cheng, L. Huang, B. Z. Du, W. Zhao, G. C. Guo, L. T. Feng, W. F. Zhang, X. F. Ren, Photonic-chip-based dense entanglement distribution. *PhotoniX* **4**, 12 (2023).

53. N. Masahiro, Y. Toshihide, N. Fumito, M. Hideaki, S. Kimikazu, High-speed avalanche photodiodes toward 100-gbit/s per lambda era. *NTT Technical Review* **16**, 45–51 (2018).

54. C. Rogers, A. Y. Piggott, D. J. Thomson, R. F. Wiser, I. E. Opris, S. A. Fortune, A. J. Compston, A. Gondarenko, F. F. Meng, X. Chen, G. T. Reed, R. Nicolaescu, A universal 3D imaging sensor on a silicon photonics platform. *Nature* **590**, 256–261 (2021).

55. W. Bogaerts, D. Pérez, J. Capmany, D. A. B. Miller, J. Poon, D. Englund, F. Morichetti, A. Melloni, Programmable photonic circuits. *Nature* **586**, 207–216 (2020).

56. S. Shekhar, W. Bogaerts, L. Chrostowski, J. E. Bowers, M. Hochberg, R. Soref, B. J. Shastri, Roadmapping the next generation of silicon photonics. *Nature Commun.* **15**, 751 (2024).

57. G. Bönsch, E. Potulski, Measurement of the refractive index of air and comparison with modified Edlén’s formulae. *Metrologia* **35**, 133–139 (1998).

58. Z. C. Wang, J. W. Zhi, H. Z. Wu, B. E. Little, S. T. Chu, J. Zhang, Z. H. Lu, C. G. Shao, W. Q. Wang, W. F. Zhang, Rapid and precise distance measurement with hybrid comb lasers. *Adv. Photonics Nexus* **3**, 046006 (2024).

59. L. Huang, W. Q. Wang, F. X. Wang, Y. Wang, C. L. Zou, L. H. Tang, B. E. Little, W. Zhao, Z. F. Han, J. Yang, G. C. Wang, W. Chen, W. F. Zhang, Massively parallel Hong-Ou-Mandel interference based on independent soliton microcombs. *Sci. Adv.* **11**, eadq8982 (2025).
